# 1,5-Bis(2-formyl­phen­oxy)-3-oxapenta­ne

**DOI:** 10.1107/S1600536808002948

**Published:** 2008-02-06

**Authors:** Nívea C. F. Dionysio, Jairo Bordinhão, Lorenzo do Canto Visentin, Célia Machado Ronconi

**Affiliations:** a Instituto de Química, Universidade Federal do Rio de Janeiro, Caixa Postal 68563, 21949-900 Rio de Janeiro, RJ, Brazil

## Abstract

In the title mol­ecule, C_18_H_18_O_5_, the two aromatic rings are connected by a flexible 3-oxapentane chain. The mol­ecule has a crystallographic twofold rotation axis (*C*_2_) passing through the central O atom. An intra­molecular C—H⋯O hydrogen bond is observed in the solid state.

## Related literature

For related literature, see: Biernat *et al.* (1992 ▶); Qi *et al.* (2005 ▶); Jeffrey & Saenger (1991 ▶); Spek (2003 ▶).
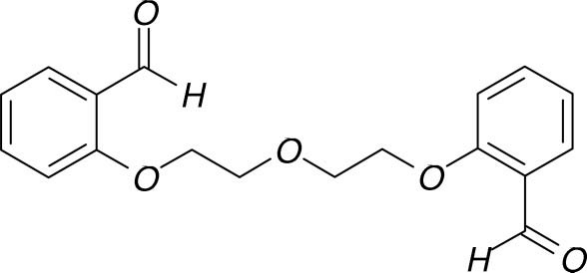


## Experimental

### 

#### Crystal data

C_18_H_18_O_5_
*M* *_r_* = 314.32
Orthorhombic, 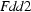

*a* = 27.613 (6) Å
*b* = 26.404 (5) Å
*c* = 4.4313 (9) Å
*V* = 3230.8 (11) Å^3^
*Z* = 8
Mo *K*α radiation
μ = 0.09 mm^−1^
*T* = 295 (2) K
0.25 × 0.08 × 0.05 mm

#### Data collection

Nonius KappaCCD diffractometer
Absorption correction: none
4449 measured reflections
1043 independent reflections
480 reflections with *I* > 2σ(*I*)
*R*_int_ = 0.134

#### Refinement

*R*[*F* ^2^ > 2σ(*F* ^2^)] = 0.051
*wR*(*F* ^2^) = 0.097
*S* = 0.99
1043 reflections
105 parameters
H-atom parameters constrained
Δρ_max_ = 0.12 e Å^−3^
Δρ_min_ = −0.14 e Å^−3^

### 

Data collection: *COLLECT* (Nonius, 1998 ▶); cell refinement: *PHICHI* (Duisenberg *et al.*, 2000 ▶); data reduction: *EVAL-14 (CCD)* (Duisenberg *et al.*, 2003 ▶); program(s) used to solve structure: *SHELXS97* (Sheldrick, 2008 ▶); program(s) used to refine structure: *SHELXL97* (Sheldrick, 2008 ▶); molecular graphics: *ORTEP-3 for Windows* (Farrugia, 1997 ▶); software used to prepare material for publication: *WinGX* (Farrugia, 1999 ▶).

## Supplementary Material

Crystal structure: contains datablocks global, I. DOI: 10.1107/S1600536808002948/si2065sup1.cif

Structure factors: contains datablocks I. DOI: 10.1107/S1600536808002948/si2065Isup2.hkl

Additional supplementary materials:  crystallographic information; 3D view; checkCIF report

## Figures and Tables

**Table 1:** Hydrogen-bond geometry (Å, °)

*D*—H⋯*A*	*D*—H	H⋯*A*	*D*⋯*A*	*D*—H⋯*A*
C9—H9⋯O2	0.93	2.42	2.764 (5)	102

**Fig. 1.**
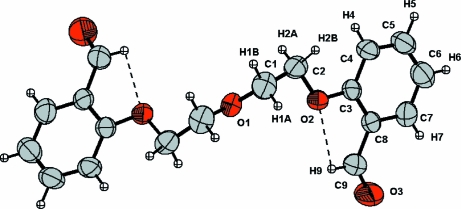
ORTEP projection of the title molecule. Symmetry equivalent atoms are generated by the symmetry code [-x, 1 - y, z]. Intramolecular bonds are indicated by dashed lines. Thermal ellipsoids are shown at the 50% probability level.

## supplementary crystallographic information

### Comment

The title molecule is generated by a symmetry operation according to the space group Fdd2. The symmetry operator (0, 1/2, *z*) applied to the equivalent atoms is a twofold rotation axis passing through the central atom O1 (Fig. 1). Bond lengths and angles are in the ranges reported for analogous structures (Biernat *et al.*, 1992; Qi *et al.*, 2005). The distances involving the C atoms in the central moiety are 1.496 (5) Å for C1—C2, 1.429 (4) Å for C1—O1, and 1.436 (4) Å for C2—O2.

The intramolecular C9—H9···O2 contact observed in the solid state (Fig. 1) is classificated as a non classical hydrogen bond (Jeffrey & Saenger, 1991). This secondary interaction does not affect the torsion angles in the 3-oxapentane chain. The torsion angle for the O2/C2/C1/O1 moiety is -69.3 (3)° and proves the flexibility of the 3-oxapentane chain. The hydrogen bonding geometry of the intramolecular contacts (Jeffrey & Saenger, 1991) with atom O2 as acceptor was calculated with *PLATON* (Spek, 2003): distances H9···O2 and C9···O2 are 2.42 and 2.764 (5) Å, respectively, and the angle at H9 is 102°.

### Experimental

A mixture of 2[2-(2-*p*-tolylsulfonyloxy)ethoxy]ethanol (1 g, 8.19 mmol), salicylic aldehyde (0.87 g, 2.10 mmol), and K_2_CO_3_ (0.47 g, 3.40 mmol) in dry MeCN (20 ml) was heated for 12 h under reflux. After the reaction mixture had cooled down to room temperature, it was filtered and the solvent removed under vacuum. The residue was purified by column chromatography (SiO_2_, Hexane:EtOAc 1:1) to give the desired product (0.55 g, 83%) as a yellow solid. A single-crystal was isolated after slow evaporation of the crystallization solvent (THF).

### Refinement

All hydrogen atoms were geometrically constrained using a riding model, with C—H distances of 0.93 Å for both benzene rings and the aldehyde moieties with *U*_iso_(H) = 1.2*U*_eq_(C*sp*^2^), and with C—H distances of 0.97 Å for the ethyl C atoms with *U*_iso_(H) = 1.2*U*_eq_(C*sp*^3^).

### Crystal data

**Table:**

C_18_H_18_O_5_	*F*_000_ = 1328
*M**_r_* = 314.32	*D*_x_ = 1.292 Mg m^−^^3^
Orthorhombic, *F**d**d*2	Mo *K*α radiation λ = 0.71073 Å
Hall symbol: F 2 -2d	Cell parameters from 50 reflections
*a* = 27.613 (6) Å	θ = 1–27.5º
*b* = 26.404 (5) Å	µ = 0.09 mm^−^^1^
*c* = 4.4313 (9) Å	*T* = 295 (2) K
*V* = 3230.8 (11) Å^3^	Plate, yellow
*Z* = 8	0.25 × 0.08 × 0.05 mm

### Data collection

**Table:**

Nonius KappaCCD diffractometer	480 reflections with *I* > 2σ(*I*)
Radiation source: fine-focus sealed tube	*R*_int_ = 0.134
Monochromator: graphite	θ_max_ = 27.5º
*T* = 295(2) K	θ_min_ = 3.1º
φ and ω scans with κ offsets	*h* = −34→35
Absorption correction: none	*k* = −34→34
4449 measured reflections	*l* = −5→5
1043 independent reflections	

### Refinement

**Table:**

Refinement on *F*^2^	Secondary atom site location: difference Fourier map
Least-squares matrix: full	Hydrogen site location: inferred from neighbouring sites
*R*[*F*^2^ > 2σ(*F*^2^)] = 0.051	H-atom parameters constrained
*wR*(*F*^2^) = 0.097	*w* = 1/[σ^2^(*F*_o_^2^) + (0.0377*P*)^2^] where *P* = (*F*_o_^2^ + 2*F*_c_^2^)/3
*S* = 0.99	(Δ/σ)_max_ < 0.001
1043 reflections	Δρ_max_ = 0.12 e Å^−^^3^
105 parameters	Δρ_min_ = −0.14 e Å^−^^3^
Primary atom site location: structure-invariant direct methods	Extinction correction: none

### Special details

**Table:**

Experimental. The transformation of the unit cell axes and *hkl* intensity data were performed by using the matrix (00–1, 010, 100) in order to solve and refine the structure in the standard setting for space group Fdd2.
Geometry. All e.s.d.'s (except the e.s.d. in the dihedral angle between two l.s. planes) are estimated using the full covariance matrix. The cell e.s.d.'s are taken into account individually in the estimation of e.s.d.'s in distances, angles and torsion angles; correlations between e.s.d.'s in cell parameters are only used when they are defined by crystal symmetry. An approximate (isotropic) treatment of cell e.s.d.'s is used for estimating e.s.d.'s involving l.s. planes.
Refinement. Refinement of *F*^2^ against ALL reflections. The weighted *R*-factor *wR* and goodness of fit *S* are based on *F*^2^, conventional *R*-factors *R* are based on *F*, with *F* set to zero for negative *F*^2^. The threshold expression of *F*^2^ > σ(*F*^2^) is used only for calculating *R*-factors(gt) *etc*. and is not relevant to the choice of reflections for refinement. *R*-factors based on *F*^2^ are statistically about twice as large as those based on *F*, and *R*- factors based on ALL data will be even larger.

### Fractional atomic coordinates and isotropic or equivalent isotropic displacement parameters (Å^2^)

**Table:**

	*x*	*y*	*z*	*U*_iso_*/*U*_eq_	
C1	0.03242 (14)	0.47183 (13)	1.1699 (9)	0.0683 (12)	
H1A	0.0138	0.4508	1.3069	0.082*	
H1B	0.0515	0.4951	1.2902	0.082*	
C2	0.06560 (13)	0.43903 (12)	0.9883 (9)	0.0607 (10)	
H2A	0.0831	0.4162	1.1207	0.073*	
H2B	0.0469	0.4188	0.8472	0.073*	
C3	0.13695 (13)	0.44746 (12)	0.6829 (8)	0.0495 (9)	
C4	0.14652 (13)	0.39549 (12)	0.6935 (9)	0.0622 (11)	
H4	0.1263	0.3740	0.8015	0.075*	
C5	0.18651 (15)	0.37623 (15)	0.5414 (11)	0.0773 (13)	
H5	0.1930	0.3417	0.5519	0.093*	
C6	0.21687 (15)	0.40706 (15)	0.3753 (10)	0.0731 (13)	
H6	0.2433	0.3935	0.2739	0.088*	
C7	0.20743 (14)	0.45820 (15)	0.3617 (9)	0.0677 (13)	
H7	0.2276	0.4791	0.2491	0.081*	
C8	0.16770 (12)	0.47928 (12)	0.5159 (10)	0.0523 (10)	
C9	0.15870 (14)	0.53391 (13)	0.4966 (13)	0.0834 (14)	
H9	0.1323	0.5466	0.6030	0.100*	
O1	0.0000	0.5000	0.9830 (7)	0.0529 (9)	
O2	0.09910 (9)	0.47057 (7)	0.8266 (6)	0.0563 (7)	
O3	0.18230 (10)	0.56356 (10)	0.3560 (9)	0.1276 (15)	

### Atomic displacement parameters (Å^2^)

**Table:**

	*U*^11^	*U*^22^	*U*^33^	*U*^12^	*U*^13^	*U*^23^
C1	0.066 (3)	0.085 (3)	0.054 (3)	−0.002 (2)	−0.005 (3)	0.018 (2)
C2	0.059 (2)	0.061 (2)	0.062 (3)	−0.007 (2)	−0.011 (2)	0.023 (2)
C3	0.044 (2)	0.045 (2)	0.059 (3)	0.0026 (19)	−0.018 (2)	0.003 (2)
C4	0.060 (2)	0.044 (2)	0.083 (3)	−0.0005 (18)	−0.018 (3)	0.0096 (19)
C5	0.075 (3)	0.047 (2)	0.110 (4)	0.010 (2)	−0.029 (3)	−0.009 (3)
C6	0.069 (3)	0.066 (3)	0.084 (3)	0.013 (2)	−0.012 (3)	−0.014 (3)
C7	0.051 (3)	0.073 (3)	0.079 (3)	0.002 (2)	−0.006 (2)	0.007 (2)
C8	0.0426 (19)	0.049 (2)	0.066 (2)	0.0020 (18)	−0.014 (2)	0.011 (2)
C9	0.059 (3)	0.053 (3)	0.138 (4)	0.004 (2)	0.011 (3)	0.023 (3)
O1	0.0469 (17)	0.066 (2)	0.0457 (19)	−0.0031 (18)	0.000	0.000
O2	0.0481 (13)	0.0462 (13)	0.0746 (18)	−0.0005 (12)	0.0020 (13)	0.0170 (14)
O3	0.090 (2)	0.0707 (18)	0.222 (4)	0.0013 (17)	0.043 (3)	0.068 (2)

### Geometric parameters (Å, °)

**Table:**

C1—O1	1.429 (4)	C4—H4	0.9300
C1—C2	1.496 (5)	C5—C6	1.381 (5)
C1—H1A	0.9700	C5—H5	0.9300
C1—H1B	0.9700	C6—C7	1.377 (4)
C2—O2	1.436 (4)	C6—H6	0.9300
C2—H2A	0.9700	C7—C8	1.407 (5)
C2—H2B	0.9700	C7—H7	0.9300
C3—O2	1.368 (4)	C8—C9	1.466 (4)
C3—C4	1.398 (4)	C9—O3	1.194 (4)
C3—C8	1.405 (4)	C9—H9	0.9300
C4—C5	1.390 (5)	O1—C1^i^	1.429 (4)
			
O1—C1—C2	111.9 (3)	C6—C5—C4	121.7 (4)
O1—C1—H1A	109.2	C6—C5—H5	119.2
C2—C1—H1A	109.2	C4—C5—H5	119.2
O1—C1—H1B	109.2	C7—C6—C5	119.1 (4)
C2—C1—H1B	109.2	C7—C6—H6	120.4
H1A—C1—H1B	107.9	C5—C6—H6	120.4
O2—C2—C1	109.1 (3)	C6—C7—C8	121.0 (4)
O2—C2—H2A	109.9	C6—C7—H7	119.5
C1—C2—H2A	109.9	C8—C7—H7	119.5
O2—C2—H2B	109.9	C3—C8—C7	119.4 (3)
C1—C2—H2B	109.9	C3—C8—C9	121.1 (4)
H2A—C2—H2B	108.3	C7—C8—C9	119.5 (4)
O2—C3—C4	124.5 (3)	O3—C9—C8	125.7 (4)
O2—C3—C8	116.1 (3)	O3—C9—H9	117.2
C4—C3—C8	119.4 (3)	C8—C9—H9	117.2
C5—C4—C3	119.5 (3)	C1^i^—O1—C1	109.1 (4)
C5—C4—H4	120.2	C3—O2—C2	117.8 (2)
C3—C4—H4	120.2		
			
O1—C1—C2—O2	69.3 (3)	C4—C3—C8—C9	179.4 (4)
O2—C3—C4—C5	−179.1 (3)	C6—C7—C8—C3	−0.8 (6)
C8—C3—C4—C5	0.7 (5)	C6—C7—C8—C9	−180.0 (4)
C3—C4—C5—C6	−1.0 (6)	C3—C8—C9—O3	−178.5 (4)
C4—C5—C6—C7	0.5 (6)	C7—C8—C9—O3	0.6 (7)
C5—C6—C7—C8	0.4 (6)	C2—C1—O1—C1^i^	176.7 (3)
O2—C3—C8—C7	−180.0 (3)	C4—C3—O2—C2	−3.0 (5)
C4—C3—C8—C7	0.2 (5)	C8—C3—O2—C2	177.2 (3)
O2—C3—C8—C9	−0.8 (5)	C1—C2—O2—C3	170.5 (3)

Symmetry codes: (i) −*x*, −*y*+1, *z*.

### Hydrogen-bond geometry (Å, °)

**Table:**

*D*—H···*A*	*D*—H	H···*A*	*D*···*A*	*D*—H···*A*
C9—H9···O2	0.93	2.42	2.764 (5)	102
